# Tuberculosis patients with diabetes co-morbidity experience reduced *Mycobacterium tuberculosis* complex clearance

**DOI:** 10.1016/j.heliyon.2024.e35670

**Published:** 2024-08-06

**Authors:** Emelia Konadu Danso, Prince Asare, Stephen Osei-Wusu, Phillip Tetteh, Amanda Yaa Tetteh, Augustine Asare Boadu, Ivy Naa Koshie Lamptey, Augustina Angelina Sylverken, Kwasi Obiri-Danso, Jane Afriyie Mensah, Abraham Adjei, Dorothy Yeboah-Manu

**Affiliations:** a Department of Bacteriology, Noguchi Memorial Institute for Medical Research, University of Ghana, Accra, Ghana; b Department of Theoretical and Applied Biology, Kwame Nkrumah University of Science and Technology, Kumasi, Ghana; c Kumasi Centre for Collaborative Research in Tropical Medicine, Kwame Nkrumah University of Science and Technology, Kumasi, Ghana; d Department of Chest Diseases, Korle-Bu Teaching Hospital, Accra, Ghana

**Keywords:** Tuberculosis, Diabetes, Treatment monitoring, TB-Molecular bacterial load assay, Bacterial clearance, And bacteria load

## Abstract

**Objective:**

This study aimed to investigate the impact of diabetes mellitus (DM) on tuberculosis (TB) treatment response using bacterial clearance as a surrogate marker.

**Method:**

We compared smear microscopy, culture, and tuberculosis molecular bacterial load assay (TB-MBLA) for treatment monitoring. Following that, bacterial clearance was longitudinally monitored among TB-only (TB without DM) and TB-diabetes (TBDM) patients using TB-MBLA.

**Results:**

Ninety-three participants, including 59 TB-only and 34 TBDM patients, were enrolled. TB-only patients exhibited higher upper zone infiltrations (32/35 vs 16/22, p = 0.059) suggesting a trend towards significance, and significantly more cavitation in the same zone (16/18 vs 7/13, p = 0.028). There was a high proportion of *Mycobacterium africanum* (Maf) among the TBDM cohort (p = 0.0044).

At baseline, TB-only patients exhibited a higher average bacterial burden (4.49 logeCFU/mL) compared to the TBDM group (3.91 logeCFU/mL) (p = 0.042). The bacterial load in the TB-only group decreased significantly during treatment but the TBDM group experienced delayed clearance throughout the intensive phase of anti-TB treatment even at day 56 (p = 0.028). The TB-only group demonstrated a shorter median time to TB-MBLA conversion to negative (57 days) compared to the TBDM group (62 days) (p = 0.022).

**Conclusion:**

These findings underscore the urgent call for understanding the interplay between diabetes and TB, emphasizing the need for tailored interventions in optimizing TB care for individuals comorbid with diabetes.

## Highlights

• TB-DM patients exhibited lower bacterial burden at diagnosis.
• There was delayed mycobacterial clearance among TB-DM patients.
• TB-only patients presented with higher upper zone infiltrations and cavitation.
• TB-DM cohort showed higher infiltrations and cavitation in middle and lower lobes.
• TB-MBLA is the most sensitive tool for assessing the response to TB chemotherapy.

## 1 Introduction

Tuberculosis caused by *Mycobacterium tuberculosis* complex (MTBC) remains a global health emergency and it is the second deadliest infectious disease caused by a single infectious agent after COVID-19. Globally, it is estimated that over 10 million new cases of TB occurred in 2022 and a quarter of these were from Africa [1]. Moreover, a worldwide estimate indicates that, 1.3 million people died of TB in 2022 [1]. The five notable risk factors of TB disease include undernourishment, HIV infection, alcohol use disorders, smoking, and diabetes mellitus (DM) [1].

In the past, TB was associated with the poor whereas DM was identified among the rich. Contrary to previous belief, DM is largely present in low- and middle-income countries where TB is most prevalent [2]. The prevalence of DM has increased over the years with an estimate of 537 adults million in 2021 according to the International Diabetes Federation (IDF). This number is expected to increase to almost 700 million by 2030 and 800 million by 2045 [3]. A systematic review and meta-analysis of sixteen observational studies in sub-Saharan Africa determined a pooled prevalence of DM among TB patients at 9.0 %. Notably, Nigeria exhibited the highest prevalence at 15 %, followed by Tanzania (11 %), and Ethiopia (10 %) [4]. Furthermore, in Ghana, Asante-Poku et al. reported a DM prevalence of 9.4 % among 2990 TB patients, contrasting with 3.3 % in the general population [5].

The standard anti-TB treatment regimen lasts for six months and includes an intensive phase of two months, during which four first-line drugs namely, isoniazid, rifampicin, pyrazinamide, and ethambutol (HRZE) are administered. This phase aims to rapidly reduce the bacterial load and prevent the spread of the disease [6]. The subsequent four-month continuation phase maintains and consolidates the treatment, usually with the administration of isoniazid and rifampicin. Overall, successful TB treatment is essential not only for the well-being of the individual but also for preventing spread of infectious particles in the community and the development of drug-resistant strains [7].

Reduction in viable bacterial load is the key biomarker for monitoring tuberculosis chemotherapy response [8]. However, microscopy which is routinely used for monitoring TB treatment response has been criticized for being less sensitive and unable to determine viability [9]. While culture detects viable bacteria, it has a longer turnaround time, prone to contamination and associated with missing information on non-culturable bacteria making it a less effective TB therapy monitoring tool and limiting the ability to take appropriate and timely decisions [9]. Ribonucleic acid (RNA) based assays have been shown to be good markers for microbial viability, thus can be used for monitoring anti-TB therapy effectively [10]. One such tool is the tuberculosis molecular bacterial load assay (TB-MBLA) which has been designed to measure the viability of *M. tuberculosis* complex through the quantification of RNA in sputum. This study therefore sought to assess the tools routinely used for monitoring TB response and use the most sensitive to determine the impact of DM on *Mycobacterium tuberculosis* complex (MTBC) clearance during anti-TB therapy.

## 2 Method

### 2.1 Study design and participants recruitment

This was an observational prospective longitudinal cohort study in which GeneXpert confirmed pulmonary TB cases aged 18 years and above were recruited between October 2020 and June 2023 from four public health facilities in the Greater-Accra Region of Ghana. All participants for this study were newly diagnosed and were recruited before the commencement of anti-TB therapy. A structured case recruitment form was used to obtain information on demographic and clinical characteristics such as cough, hemoptysis, infiltration, HIV, lesion location, patient's demographics, anthropometrics, social history, previous history regarding the use of anti-TB medication, symptoms such as polydipsia, polyphagia, and polyuria. Furthermore, lesion characteristics were determined by digital X-ray.

In accordance with the American Diabetes Association (ADA) criteria, a finger-prick blood (using ACCU-CHEK Guide Test Strips) was used to assess the random blood glucose (RBG) levels of all recruited study participants using a glucometer (ACCU-CHEK, Roche Diabetes Care Limited, Burgess Hill, UK). Patients with RBG≥7 mmol/L had their blood drawn for a confirmatory test (glycated hemoglobin; HbA1c). Patients with HbA1c ≥ 6.5 % were classified as diabetic (TBDM) cohort and those with HbA1c<6.5 % were TB-only cohort. All the TBDM patients were on anti-DM treatment except 3 and all those who were being treated for diabetes were on metformin. All samples obtained from patients were properly labeled and the data was recorded in Microsoft Access office 365 for analysis.

### 2.2 Inclusion and exclusion criteria

TB-only cohort refers to newly diagnosed TB cases above 18 years with no known co-infection or immune suppression illness, or not on immunosuppressive drugs that predispose one to TB and who consented to the study.

TBDM cohort was made up of participants with co-morbidity of TB and DM who consented to the study. Children below the ages of 18 years, HIV/AIDS patients, individuals on immunosuppressive drugs, pregnant women and participants who did not give consent were excluded. Patients with incomplete data, negative TB-MBLA and culture results, lost to follow-up, defaulted and those who died before 2 months of treatment were excluded from the bacterial clearance analysis.

### 2.3 Monitoring of patients during anti-TB treatment

Per the national tuberculosis control program protocol, serial sputa were collected from a section of the participants (TB-only and TBDM concurrently) at different timepoints. These were monitored during anti-TB treatment (follow-up) using clinical and microbiological indicators. Clinical observations included monitoring vital signs such as weight, body mass index (BMI), blood sugar levels, and blood pressure. Microbiological investigations using early morning serial sputum samples were conducted to assess treatment response. DM was managed by the patient's clinicians per their routines. Time of recovery was documented, all treatment adverse conditions were also noted, eg. vomiting, edema, peripheral neuropathy, headache, myalgia, etc. HbA1c levels were determined for all patients and retested after 3 months of TB treatment. Adherence to medication was rigorously assessed throughout the study using a combination of self-reported adherence measures, and pill counts from the public health yellow card.

Participants were also counselled on the importance of adhering to medications (both anti-TB and anti-diabetes drugs), ensuring good ventilation, adopting a healthy diet and lifestyle, and recognizing the need for follow-up appointments. Patients were educated to promptly report any unusual symptoms to the health facility before their scheduled review date.

### 2.4 Assessment of mycobacterial clearance dynamics

Serial sputum samples were collected from all participants on the following time points: Days 0, 3, 7, 14, 28 and 56 as well as days 84, 112, 140 and 168 provided there was a productive cough.

**Culture:** Sputum samples were decontaminated with equivalent volumes of 5 % (w/v) oxalic acid and later inoculated on two pairs of Lowenstein-Jensen (LJ) media slants supplemented with glycerol and pyruvate in separate glass tubes. The inoculated tubes were incubated at 37 °C and observed weekly for confluent mycobacteria growth for 3 months [11].

**Smear Microscopy:** Sputum smears were prepared directly from the decontaminated resuspended pellet on clean well-labeled microscope slides and stained using the Ziehl-Neelsen staining method. The stained slides were then examined under the microscope and quantified using a grading system earlier described [12].

### 2.5 Identification of *Mycobacterium tuberculosis* complex lineages

Mycobacteria isolates were harvested, and DNA was extracted by boiling isolates at 95 °C for 1 h to release mycobacterial nucleic acid material into the supernatant. The supernatant of the heat killed isolates was used for further molecular analysis. The obtained nucleic acid of the isolates was initially confirmed as members of the MTBC by amplification of the insertion sequence *6110* (IS*6110*) using polymerase chain reaction (PCR). The confirmed isolates were then genotyped using spacer oligonucleotide typing (spoligotyping) by amplification of the direct repeat region and subsequent hybridization onto a film [13]. The obtained binary data indicating the presence or absence of spacers in the DR region were analyzed in the MIRU-VNTR*plus* database to determine the infecting lineages and sub-lineages [14].

**TB-Molecular Bacterial Load Assay:** The quantification of *M. tuberculosis* was achieved using TB-MBLA as described previously [15]. Briefly, serial sputa samples preserved in Guanidine thiocyanate (GTC) collected at different time points were processed for RNA extraction using the FastRNA Pro Blue Kit and internal control was added to each sample, followed by centrifugation and removal of GTC supernatant. Lysis buffer was added to the pellets, which were then homogenized using a homogenizer. The aqueous phase was obtained, and RNA was precipitated with ice-cold ethanol. After drying, the RNA was treated with Turbo DNase and stored at −80 °C. The QuantiTect Multiplex RT-PCR NR Kit (QT) (Qiagen Inc., Hilden, Germany) was modified with specific primers and probes for MTBC 16S ribosomal RNA. Duplicate analysis of RNA from each sample, along with extraction controls and known RNA concentrations, allowed the construction of standard curves. These curves served as a reference for converting Cq values into bacterial load, measured as estimated colony forming units per milliliter (eCFU/mL). The threshold indicating positivity for TB-MBLA was set at a 30 Cq value, aligning with a corresponding 1.0 logeCFU/mL. Results surpassing this cutoff were deemed negative in the test evaluation.

### 2.6 Statistical analysis

The participants' information collected during recruitment and treatment was double entered into Microsoft Access office 365 as well as cross checking the electronic data with the structured questionnaire to eliminate data entry inconsistencies and duplications. Data analysis was done using graph pad prism version 9.4.1 and Stata version 14.2. Statistical comparisons were computed using Fisher's exact tests and Chi-square test as appropriate with p < 0.05 set as the level of significance.

## 3 Results

### 3.1 Clinical and demographic characteristics of study participants

**Fig. 1:**
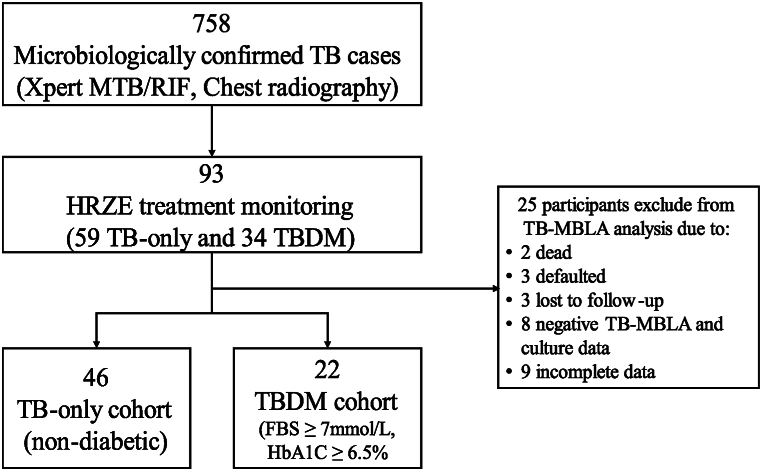
Continuum of longitudinal study participants and distribution among cohorts.

The characteristics of the study participants are presented in Table 1. This study is part of a bigger study that recruited 75 (9.9 %) TBDM out of 758 GeneXpert newly diagnosed pulmonary TB patients. Ninety-three participants comprising 59 (63.4 %) TB-only and 34 (36.6 %) TBDM were recruited for this longitudinal study. HbA1c of the TBDM cases ranged from 6.9 to 14.4 % and all had uncontrolled diabetes. Reasons for exclusion and patient's distribution are outlined in Fig. 1. Statistical analysis using chi-squared test shows that there was no significant difference between the excluded participants and the cohort we carried forward for further analysis, [tabi 46 22 \ 13 12, chi (p = 0.165)], consequently, we did not expect any significant bias in our downstream analysis. All the 93 cases, except one (TB-only), were found to be susceptible to rifampicin as per the GeneXpert results. Consistent with TB epidemiology in Ghana, majority of participants were males, 63 (67.7 %). Their median (IQR) age was 45 years (28–56). Generally, TBDM individuals were older (55.5 years: IQR: 46–60) than the TB-only (32 years: IQR: 24–49) cohort (p < 0.001). Out of the 93 cases, over a half of them (60, 64.5 %) consume alcohol on regular basis. Clinical symptoms did not differ among the two groups. Both cohorts presented with high levels of abnormal X-ray results. TB-only patients exhibited higher upper zone infiltrations (32/35 vs. 16/22; p = 0.059), suggesting a trend towards significance, and significantly more cavitation in the same zone (16/18 vs. 7/13; p = 0.028). However, infiltration in the lower lobe (62.9 % vs 63.6 %, p = 0.953) and middle lobe (80.0 % vs 86.4 %, p = 0.539), as well as cavitation in the lower lobe (11.1 % vs 23.71, p = 0.371) and middle lobe (38.9 % vs 46.2 %, p = 0.686), were not significantly different between TB-only and TBDM patients. The most common symptoms were weight loss (95.7 %), extreme cough (82.8 %) and chest pains (75.3 %) (Table 1).

**Table 1:** Demographical and clinical characteristics of participants.

Variables	Overall (N = 93) N (%)	TB-only (n = 59) n, (%)	TBDM (n = 34) n, (%)	p-value
Gender (Male)	63 (67.7)	43 (68.3)	20 (31.7)	0.163
Age (Years)				
15-25	16 (17.2)	16 (27.1)	0 (0.0)	<0.001
26-40	30 (32.3)	23 (39.0)	7 (20.6)	
41-60	32 (34.4)	13 (22.0)	19 (55.9)	
Over 60	15 (16.1)	7 (11.9)	8 (23.5)	
TB status (n = 91)				
New case	85 (93.4)	55 (94.8)	30 (90.9)	0.325
Relapse	6 (6.6)	3 (5.2)	3 (9.1)	
Alcohol use (Yes)	60 (64.5)	39 (66.1)	21 (61.8)	0.674
Smoking (Yes)	26 (28.0)	18 (30.5)	8 (23.5)	0.470
Mycobacterium burden at diagnosis (n = 91)				0.637
MTB RIF-high	58 (63.7)	36 (63.2)	22 (64.7)	
MTB RIF-medium	20 (22.0)	14 (24.6)	6 (17.7)	
MTB RIF-low	13 (14.3)	7 (12.3)	6 (17.7)	
Chest X-ray (n = 60)	58 (96.7)	36 (94.7)	22 (100)	0.274
Abnormal				
Cavitation (n = 31)				
Upper zone	23 (74.2)	16 (88.9)	7 (53.9)	0.028
Middle zone	13 (41.9)	7 (38.9)	6 (46.2)	0.686
Lower zone	5 (16.1)	2 (11.1)	3 (23.1)	0.371
Infiltration (n = 57)				
Upper zone	48 (84.2)	32 (91.4)	16 (72.7)	0.059
Middle zone	47 (82.5)	28 (80.0)	19 (86.4)	0.539
Lower zone	36 (63.2)	22 (62.9)	14 (63.6)	0.953
Clinical Signs				
Night Sweat	61 (65.6)	37 (62.7)	24 (70.6)	0.441
Chest Pains	70 (75.3)	47 (79.7)	23 (67.7)	0.196
Weight Loss	89 (95.7)	58 (98.3)	31 (91.2)	0.103
Cough (>2 weeks)	77 (82.8)	49 (83.1)	28 (82.4)	0.932
Dyspnoea	50 (53.8)	31 (52.5)	19 (55.9)	0.756
Hemoptysis	8 (8.6)	3 (5.1)	5 (14.7)	0.111
Fever	26 (28.0)	17 (28.8)	9 (26.5)	0.808
Myalgia	26 (28.0)	17 (28.8)	9 (26.5)	0.808

### 3.2 Characterization and distribution of MTBC

Infecting genotypes were defined for over 90 % of the cases recruited for this longitudinal study and out of these, 80 % were monitored throughout the standard HRZE TB chemotherapy.

Of the 93 cases followed, 78 (83.9 %), 81 (87.1 %) and 85 (91.4 %) were microscopy (acid fast bacilli), culture and TB-MBLA positive respectively at baseline. Based on spoligotyping, we identified 4 out of the 9 humans-adapted MTBC lineages (L) (L1, L4, L5, L6) among TB only population and L4, L5 and L6 among the TBDM group. We obtained TB spoligotypes for 85 comprising 65 (76.4 %) *Mycobacterium tuberculosis* sensu stricto (MTBss) (L1 and L4) and 17 (20.0 %) *Mycobacterium africanum* (Maf: that is, L5 and L6). Our collection also included 1 (1.2 %) animal strain (*M. bovis*) and 2 (2.4 %) orphan (new) strains (Table 2). There was a high proportion of Maf among the TBDM cohort, L6 [TB-only: 4/56 (7.1 %) vs TBDM: 6/29 (20.7 %)) and L5 (TB-only: 2/56 (3.6 %) vs TBDM: 5/29 (17.2 %)]

**Table 2:** Infecting genotype distribution.

	Infecting genotypes	Total	TB-only n (%)	TBDM n (%)	p-value
MTBss	L1	2	2 (3.7)	0 (0.0)	0.0044
	L4	63	46 (85.2)	17 (60.7)	
Maf	L5	7	2 (3.7)	5 (17.9)	
	L6	10	4 (7.4)	6 (21.4)	
**Total**		**82**	**54**	**28**	

(OR = 5.18, 95 % CI 1.70 to 17.19, p = 0.0044) (Table 2).

Data analyses for the study were carried out on 93 participants undergoing TB treatment. Out of the 93 participants longitudinally monitored, 25 were excluded for reasons detailed in the figure. For the two patients who died, one was TB-only and the other was TBDM. The bacterial load at diagnosis for both were high.

Table 1 describes the demographic and clinical details of the study participants. MTB and RIF refer to *Mycobacterium tuberculosis* and rifampicin respectively. The interpretation of the Ct values for mycobacterium burden is as follows: MTB RIF-high is less than 16, MTB RIF-medium ranges from 16 to 22, and MTB RIF-low ranges from 23 to 28.

Table 2 shows the distribution of infecting genotypes based on SNP typing and lineages using spoligotyping. L depicts lineage, L1-L6 is lineage 1–6 respectively. MTBss: *Mycobacterium tuberculosis* sensu stricto and Maf: *Mycobacterium africanum*. N = 82: TB-only = 54 and TBDM = 28.

### 3.3 Performance of microbiological tools for monitoring anti-TB therapy

**Fig. 2:**
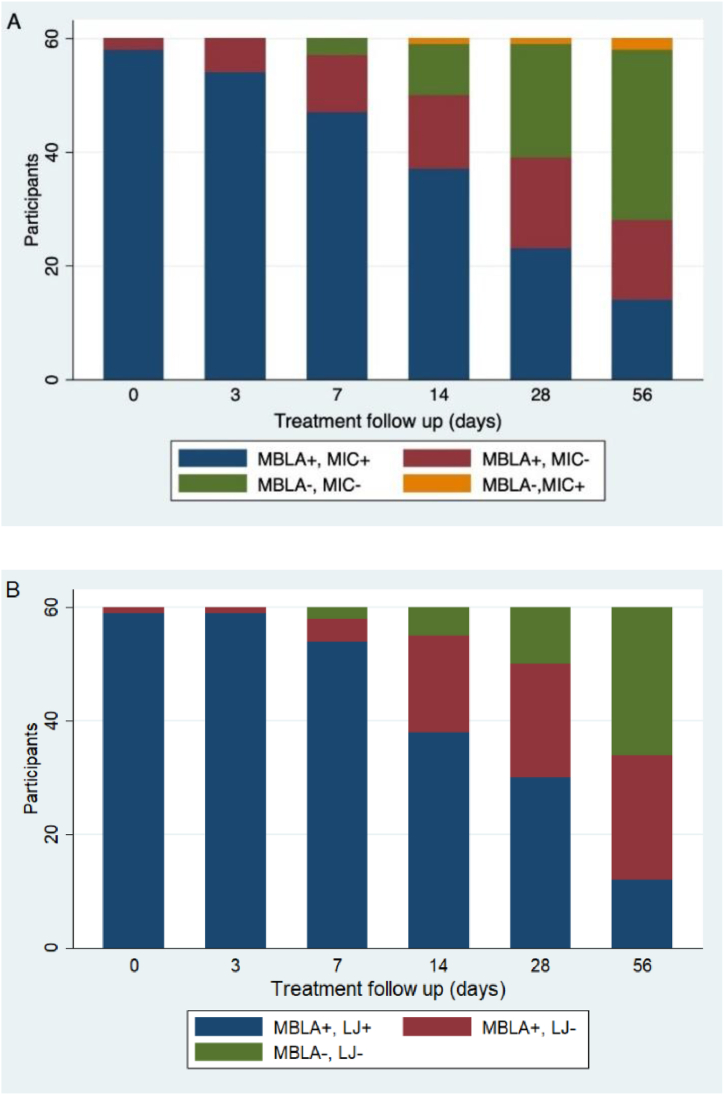
Comparing the sensitivity of traditional methods for monitoring TB chemotherapy (standard RHZE) response during the intensive phase (Day 0 to Day 56). A: Comparing MBLA to smear microscopy and B: Comparing MBLA to mycobacteria culture. MBLA+, MIC + denotes the number of patient that were positive by MBLA and smear microscopy, MBLA+, MIC- denotes the number of patients that were positive by MBLA but negative by smear microscopy, MBLA-,MIC- denotes the number of patients who converted to negative by TB-MBLA and smear microscopy and MBLA-, MIC + denotes the number of patients who converted to negative by TB-MBLA but positive by smear microscopy, N = 60.

The *Mycobacterium tuberculosis* (MTB) burden measured by TB-MBLA, mycobacteria culture (Lowenstein-Jensen) (LJ) and smear microscopy (MIC) (Fig. 2) decreased significantly over time (p < 0.001). Both A (MIC vs TB-MBLA) and B (LJ vs TB-MBLA) showed similar trends where almost all the cases were reported as positive by all three methods at day 0. However, throughout treatment, a proportion of cases (shown in red) were reported as negative by both smear microscopy (D0: 3.3 %, D3: 10 %, D7: 16.7 %, D14: 21.7 %, D28: 26.7 % and D56: 23.3 %) and mycobacteria culture (D0: 1.7 %, D3: 1.7 %, D7: 6.7 %, D14: 28.3 %, D28: 33.3 % and D56: 36.7) (MBLA+, MIC- or MBLA+, LJ-) but were correctly detected as positive by TB-MBLA especially, cases with lower bacterial load (scanty). A small proportion (2 cases) was reported as positive (non-viable mycobacteria) by microscopy and negative by TB-MBLA (shown in yellow) (Fig. 2A). TB-MBLA was found to be the most efficient tool for monitoring TB chemotherapy response compared to microscopy and culture (Fig. 2).

### 3.4 Comparing *Mycobacterium tuberculosis* clearance among TB-only and TBDM participants

**Fig. 3:**
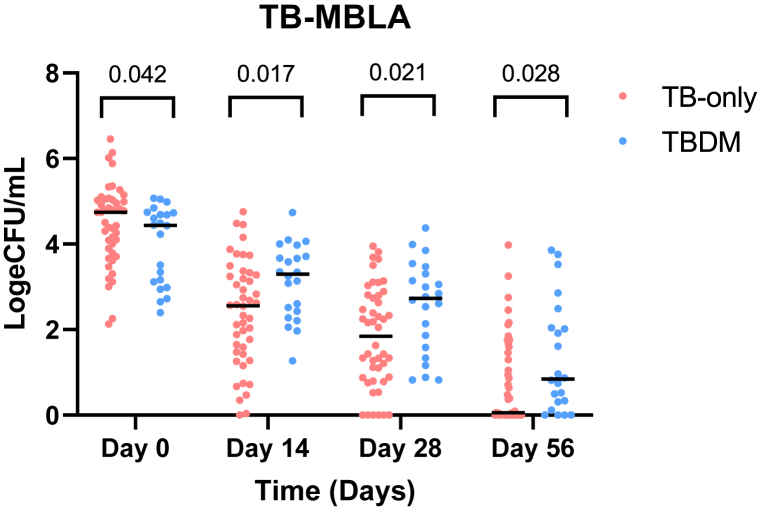
Assessment of mycobacterial clearance among cohorts using TB-MBLA. Dot plots show diversity of mycobacterial clearance among cohorts during anti-TB intensive phase (Day 0 to Day 56) of chemotherapy (RHZE). Each dot represents a case. N = 68: TB-only = 46, TBDM = 22.

**Fig. 4:**
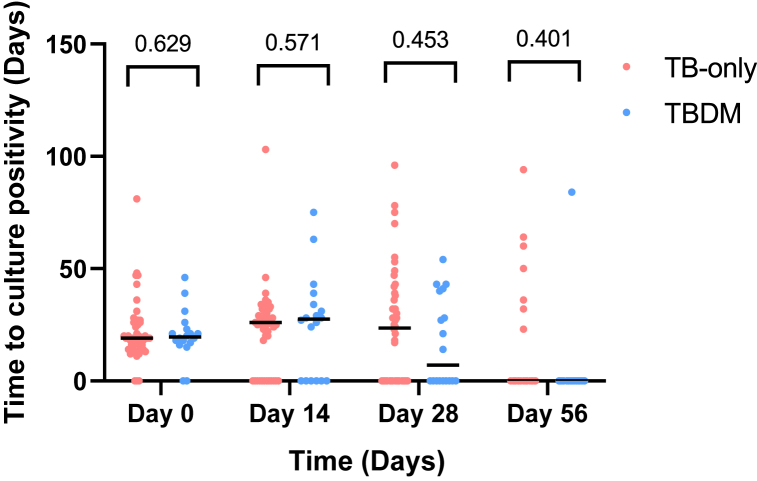
Comparison of time to culture positivity among cohorts using TB-MBLA. Dot plot shows the number of days from time of culture to the time first macroscopic growth was obtained during anti-TB intensive phase (Day 0 to Day 56) of chemotherapy (RHZE). Each dot represents a case. N = 65: TB-only = 48, TBDM = 17.

The logeCFU/mL measured by TB-MBLA decreased significantly with time but the MTBC reduction rate varied (Fig. 4) among TB-only and TBDM cohorts. The mean MTBC load in logeCFU/mL (95 % CI) was reduced from 4.49 (4.22–4.75) at baseline to 2.43 (2.07–2.78) at day 14, then to 1.86 (1.50–2.20) at day 28 and 0.73 (0.43–1.02) at day 56 for TB only group. For the TBDM group, MTB burden reduced from 3.91(3.48–4.34) at baseline to 3.12 (2.71–3.53) at day 14, then to 2.51(1.98–3.00) at day 28 and 1.41 (0.79–2.03) at day 56 through anti-TB treatment. At baseline, the average bacterial burden among TB-only patients was observed to be higher compared to the TBDM group (p = 0.042) and after, the bacterial load among TB-only group reduced appreciably. Despite the lower bacterial burden observed at diagnosis, the TBDM group delayed in clearance through anti-TB treatment (day 14: p = 0.017, day 28: p = 0.021) even at day 56 (p = 0.028). The initial TB-MBLA conversion occurred on day 7 and 14 among TB-only and TBDM groups respectively (difference of seven days). Further analysis using mixed-effects model also showed a significant difference between the cohorts over time (p = 0.0004) with correlation coefficient of 0.92 (95 % CI: −0.02 to 0.99, p = 0.04) (Fig. 3).

### 3.5 Comparison of time to culture positivity between TB-only and TBDM participants

Bacterial load was further assessed by comparing the time to culture positivity between the two cohorts using TB-MBLA. The higher the bacterial load, the lower the time to culture positivity. This study showed no significant difference in the time to culture positivity between the two cohorts at the various time points (Fig. 4). A mixed-effects analysis also showed no significant difference between the time to culture positivity for TB-only and TBDM cohorts over time (p = 0.640) with a correlation coefficient of 0.91 (95 % CI: −0.38 to 0.99, p = 0.085).

### 3.6 Comparison of *Mycobacterium tuberculosis* clearance duration among cohorts

**Fig. 5:**
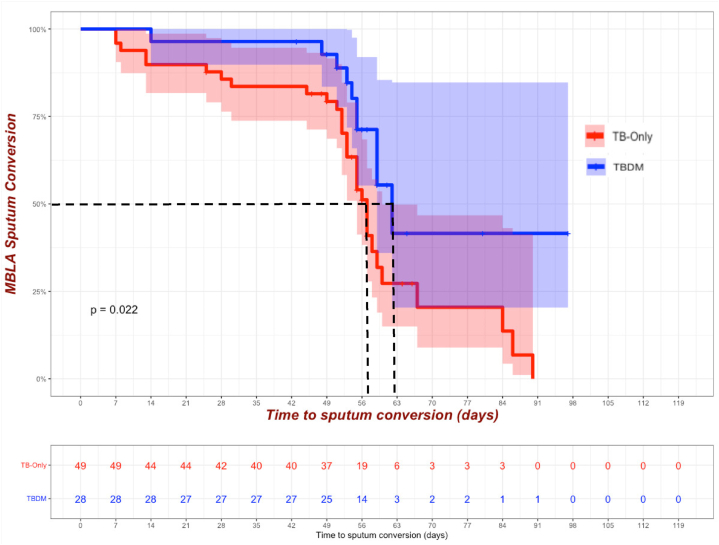
Kaplan-Meier curves showing median time to *Mycobacterium tuberculosis* killing in patients' sputum per cohort. The dotted lines denote the median time to TB-MBLA conversion from positive to negative. N = 68: (TB-only = 46, TBDM = 22).

The highest *M. tuberculosis* killing rate was observed in a TB-only case (Fig. 5) followed by a TBDM case. The TB-only group had shorter (p = 0.022) median time to TB-MBLA conversion to negative compared to the TBDM group, 57 vs 62 days respectively.

## 4 Discussion

Assessing a patient's response to anti-TB treatment holds immense significance for global TB control efforts. It aids in the identification of patients who may be at risk of treatment failure, especially among individuals with multimorbidity such as diabetes and HIV. Our study is pivotal in addressing queries related to treating individuals with DM and TB. Furthermore, this study will contribute to tools for monitoring TB treatment and ultimately early detection of treatment failure as well as drug resistance. Our main objective was to assess the impact of DM on TB care and we used bacterial clearance as a surrogate for treatment response. But to do this we needed an effective and rapid tool to measure bacterial clearance.

Results from this research affirm the perspective that TB-MBLA, which measures gene expression stands out as a sensitive tool for assessing the response to TB chemotherapy. TB-MBLA demonstrated its efficiency by detecting a greater number of cases at each time point that might have been classified as negative by the traditional methods during the intensive phase of TB treatment [8,16]. TB-MBLA does not only have the capability to detect and quantify viable replicating MTBC in patients' sputum, but it is also efficient in quantifying dormant MTBC, thereby preventing data loss. This efficiency enables the precise determination of bacterial burden in patients' sputum. The turnaround time for TB-MBLA is significantly shorter (24 h) compared to mycobacterial culture methods for detecting viable bacteria [16].

The expedited decision-making process is crucial in reducing drug resistance, avoiding unnecessary expenses related to patients' medication and hospital stays or admissions [8]. For instance, in this current study, a participant with slow bacterial clearance that was detected by only TB-MBLA at day 56 was later found to be isoniazid resistant. Subsequently, the treatment of the patient was modified by the clinician to address the delayed clearance. Identifying such cases is crucial for appropriate treatment regimen and reducing drug resistant TB.

This study demonstrated that DM serves as an independent risk factor contributing to the delayed clearance of mycobacteria during the intensive phase of conventional TB therapy. This phase is crucial for rendering the patient non-infectious [17].

Protective immune response against MTBC primarily relies on cell-mediated immunity, orchestrated by specialized immune cells such as macrophages, T lymphocytes, CD4^+^ T cells and CD8^+^ T cells [18]. However, the hyperglycemic state in DM could compromise the competency of the immune cells, impairing their normal function [19]. The formation of granulomas, organized structures encapsulating infected cells, is critical in controlling latent TB infection. CD4^+^ T cells contribute to this process, preventing the reactivation of latent TB infection [20]. This immune dysfunction may render individuals with diabetes more vulnerable to severe TB manifestations and challenges in treatment outcomes including slow bacterial clearance [19]. Furthermore, the hyperglycemic state in TBDM patients could create an environment conducive for microbial growth, potentially affecting the rate of mycobacterial clearance.

According to our radiographic observations, individuals with comorbid TB and diabetes exhibited higher occurrences of middle and lower lung involvement (Table 1). This observation could be confirmatory for the role of delayed production of IFN-γ that could lead to increased inflammation, contributing to the development of more extensive lung lesions, cavitation, and infiltration that extends into multiple lobes [21]. Another plausible explanation for this pattern may be rooted in the elderly and diabetic population, where elevated alveolar oxygen pressure in the lower lobes facilitates disease progression in these regions [22].

On the other hand, the slow bacterial clearance during treatment in TBDM cases could stem from the documented alterations in rifampicin metabolism among TBDM patients [19,23]. This outcome may be attributed to a decline in the gastrointestinal absorption of ingested rifampicin or an expansion in volume distribution resulting from the greater body weight observed in TBDM patients [24]. Consequently, it is imperative to conduct a prospective assessment of personalized dosing for isoniazid and rifampicin, considering plasma concentration measurements [24]. To comprehend the suboptimal levels of TB drug concentration in patients' blood during treatment, an ongoing component of the current study is investigating pharmacological interactions.

At the time of diagnosis, TBDM patients exhibited a lower bacterial burden compared to individuals with TB without DM. The lower bacterial burden observed in TBDM Patients is comparable to other immunocompromised TB comorbidities such as TB-HIV patients who mostly present with paucibacillary TB. Therefore, advanced diagnostic tools such as TB-MBLA, become crucial for accurate and timely diagnosis [25]. Delayed diagnosis contributes to disease progression, increases the risk of severe clinical manifestations and transmission [26].

This study establishes Maf as an opportunistic pathogen, particularly in diabetic TB patients who display increased susceptibility to Maf infection. This aligns with findings from prior research by Asante-Poku et al. and de Jong et al., linking Maf L6 to HIV and its higher prevalence among older TB patients. Geographically confined to West Africa, where diabetes prevalence is rising, Maf is contributing significantly to pulmonary TB cases, reaching up to 40 % in some West African countries and 20 % in Ghana [5,27].

We recommend future studies to consider recruiting larger cohort sample sizes to enable the use of logistic regression models to enrich data analysis. The strength of this study is the ability to follow patients through treatment to monitor the influence of DM on TB chemotherapy. In conclusion, this study provides significant insights into the complex interplay between TB and DM, shedding light on several key findings. Our findings do not only add valuable data to the limited pool of information on TBDM interactions but also underscore the urgency of considering comorbidities in TB management strategies. The findings advocate for enhanced screening measures for both TB and DM in affected populations, offering a crucial step forward in addressing the dual burden of these diseases, particularly in regions experiencing a rise in diabetes prevalence.

## Funding

This work was funded by the 10.13039/501100001713European and Developing Countries Clinical Trials Partnership (10.13039/501100001713EDCTP) Senior Fellowship to DY-M (TMA.2017.GSF.1942-TB-DM). Dorothy Yeboah-Manu is a GSK-EDCTP Senior Fellow.

## Ethical approval statement

Ethical approval for all study protocols were obtained from the Noguchi Memorial Institute for Medical Research Institutional Review Board and Korle-Bu Teaching Hospital Institutional Review Board both in Ghana. Informed consent was sought from each confirmed TB case before enrolment as a participant in the study.

## Data availability statement

Data associated with this study has not been deposited into a publicly available repository, however, all data generated from this study have been included in this article/supp. material/referenced in article.

## CRediT authorship contribution statement

**Emelia Konadu Danso:** Writing – review & editing, Writing – original draft, Methodology, Investigation, Formal analysis, Data curation, Conceptualization. **Prince Asare:** Writing – review & editing, Writing – original draft, Project administration, Formal analysis, Data curation. **Stephen Osei-Wusu:** Writing – review & editing, Writing – original draft, Methodology, Investigation, Formal analysis. **Phillip Tetteh:** Writing – review & editing, Writing – original draft, Methodology, Data curation. **Amanda Yaa Tetteh:** Writing – review & editing, Writing – original draft, Methodology, Data curation. **Augustine Asare Boadu:** Writing – review & editing, Writing – original draft, Data curation. **Ivy Naa Koshie Lamptey:** Writing – review & editing, Writing – original draft, Data curation. **Augustina Angelina Sylverken:** Writing – review & editing, Writing – original draft, Supervision. **Kwasi Obiri-Danso:** Writing – review & editing, Writing – original draft, Supervision. **Jane Afriyie Mensah:** Writing – review & editing, Writing – original draft, Investigation. **Abraham Adjei:** Writing – review & editing, Writing – original draft, Investigation. **Dorothy Yeboah-Manu:** Writing – review & editing, Writing – original draft, Visualization, Supervision, Resources, Project administration, Methodology, Funding acquisition, Conceptualization.

## Declaration of competing interest

The authors declare that they have no known competing financial interests or personal relationships that could have appeared to influence the work reported in this paper.
